# New epidemiological profile of schistosomiasis from an area of low prevalence in Brazil

**DOI:** 10.1590/0037-8682-0335-2020

**Published:** 2020-10-21

**Authors:** Israel Gomes de Amorim Santos, Letícia Pereira Bezerra, Tatyane Martins Cirilo, Laryssa Oliveira Silva, João Paulo Vieira Machado, Pedro Dantas Lima, Martha Rejane Souza Bispo, Sheilla da Conceição Gomes, Glória Isabel Lisboa da Silva, Vitória Jordana Bezerra Alencar, Ivisson Abreu Damasceno, Mikaelly Maria Vieira de Carvalho, Dharliton Soares Gomes, Rosália Elen Santos Ramos, Edmilson Genuíno Santos, Luiz Carlos Alves, Fábio André Brayner

**Affiliations:** 1 Fundação Oswaldo Cruz, Instituto Aggeu Magalhães, Programa de Pós-Graduação Stricto Sensu em Biociência e Biotecnologia em Saúde, Recife, PE, Brasil.; 2 Universidade Estadual de Alagoas, Departamento de Biologia, Santana do Ipanema, AL, Brasil.; 3 Universidade Federal de Sergipe, Programa de Pós-Graduação Stricto Sensu em Biologia Parasitária, São Cristóvão, SE, Brasil.; 4 Universidade Federal de Pernambuco, Laboratório de Imunopatologia Keizo Asami, Recife, PE, Brasil.

**Keywords:** Epidemiology, Parasitic diseases, Schistosoma mansoni, Spatial analysis

## Abstract

**INTRODUCTION:**

Schistosomiasis, caused by infection from *Schistosoma mansoni,* is a disease that represents an important public health problem for Brazil, especially for states in the Northeast region. Thus, the aim of this study is to present a new epidemiological profile for the disease in a municipality with low prevalence in the state of Alagoas, Brazil.

**METHODS:**

A cross-sectional study was conducted through a coproparasitological and malacological survey. A structured questionnaire was applied to the study participants to survey possible risk factors and a spatial analysis (kernel density) was used to measure the risk of infection.

**RESULTS:**

Of the 347 participants, 106 (30.5%) were infected by *Schistosoma mansoni*, most of them from the urban area of the municipality (68.9%; 73/106). A 3-fold risk of infection was found for individuals living in the urban area and a risk of 2.15 times for self-declared farmers. *Biomphalaria glabrata* and *B. straminea* were the species found in the municipality, but no animals were diagnosed as infected by the parasite. Spatial analysis showed a random distribution of vectors and human cases of the disease, and the formation of two clusters of human cases in the urban area was seen.

**CONCLUSIONS:**

A new epidemiological profile for schistosomiasis from *S. mansoni* infection was presented in a municipality of low endemicity: a high proportion of positive individuals in the urban area; presence of snails without positive diagnosis for *S. mansoni* infection; random distribution of vectors and human cases; and absence of association between classical risk factors and human infection.

## INTRODUCTION

Schistosomiasis, a disease caused by the trematode worm *Schistosoma mansoni*, continues to be a major problem for Brazilian public health. The last survey of the disease, conducted in the country from 2010 to 2015, showed a significant reduction in positive cases of the disease^1^, but studies have shown that the real epidemiological situation of the disease is underestimated^1^ ^-^ ^4^, mainly due to the inefficiency of the method of parasitological analysis used in coproscopic surveys^5^ ^,^ ^6^.

In addition to the inefficiency of the diagnostic method, the last survey also showed that the historically endemic areas of the country continue with their epidemiological status and are of great relevance to the disease. These areas are located predominantly in the northeast of the country and belong to the states of Pernambuco, Sergipe, and Alagoas, which demonstrates that the endemic disease remains recrudescent in these states, despite all the control measures adopted and all the studies conducted^1^.

In Alagoas, in particular, data from the Information System of the Schistosomiasis Control Program (SISPCE)^7^ show that there are municipalities where the positivity rate of the disease exceeds 20%, but most municipalities in the state have positivity rates that make them municipalities of low endemicity for the disease. This epidemiological scenario of municipalities with low endemicity was also found to be the case for most of the municipalities evaluated in all states in the national survey.

In these municipalities of low endemicity, especially in the Northeast, the epidemiological profile that characterizes them is the greatest prevalence of the disease in rural areas, with male individuals being the most affected. Additionally, infected intermediate hosts of the pathogen, *Biomphalaria glabrata* and/or *B. straminea*, are present in the municipality and eliminate cercariae. Finally, an overlap exists between the presence of infected intermediate hosts and human cases positive for schistosomiasis^8^ ^,^ ^9^.

Thus, considering the studies that show a pattern in the epidemiological link in areas of low prevalence for schistosomiasis, with the knowledge that the diagnostic method used by the teams of the Schistosomiasis Control Program (PCE) may underestimate the real prevalence of the disease in areas of low endemicity, the objective of this study is to present a new epidemiological profile for schistosomiasis caused by *S. mansoni* infection in a municipality of low endemicity in the state of Alagoas, Brazil.

## METHODS

### Study Location

The study was performed in the city of Lagoa da Canoa, Alagoas, Brazil. This city is located in the Agreste region of the state and has an estimated population of 17,852 inhabitants. About half of the population resides in the urban area of the city and the other half in the rural area. Agriculture is the predominant economic activity in the municipality, with tobacco and cassava crops being the main source of income within this economic category^10^ for both urban and rural dwellers.

In relation to schistosomiasis, the municipality is one of the 70 municipalities of the state of Alagoas endemic for the parasitosis, with its prevalence estimated at about 8.06% in a 10-year analysis of data from the Information System of the Schistosomiasis Control Program^7^. Thus, according to the criteria adopted by Brazilian Ministry of Health, the municipality is considered of low endemicity for schistosomiasis mansoni.

### Study Design

This is an analytical study, with a cross-sectional approach of human and malacological cases collected in the city of Lagoa da Canoa, Alagoas, Brazil.

### Parasitological Survey

A population-based parasitological survey was conducted. To define the number of individuals participating in the study, the following parameters were used: 3% sampling error, the assumed prevalence of SISPCE data (8.06%)^7^, and the population estimated by the IBGE census of 2010 (18,250 inhabitants)^11^. The minimum number of participants was estimated as 311 individuals, but, considering the losses, it was decided to add 20% more individuals to (n), which raised the number of participants to 373.

Individuals were randomly selected from the urban and rural areas, from all over the city, in proportion with the individuals belonging to both zones. To this end, the number of individuals assisted by each Family Health Program was collected at the municipal health department and a proportional distribution, within each Family Health Program, of the number of people assisted by each community health agent in the unit’s area of coverage was carried out. In the urban area, from the first house where individuals accepted to participate in the study, the other residences were chosen within an interval of between 50 and 100 meters of distance from one residence to another. In the rural area, a sketch was used to help randomize the collections. In both areas, community health workers helped to approach the families. 

In the municipality 9,165 (50.2%) individuals are from the urban area and 9,085 (49.8%) individuals are from the countryside^12^. Thus, the final sample consisted of 174 individuals from the urban area and 173 individuals from the rural area. From each study participant, 3 samples of fecal material were collected every other day and, from each sample, 2 slides were prepared by the Kato-Katz method. Each slide was read by 2 different analysts, and a third analyst was consulted when the results of the first evaluators differed by more than 30% in the account of the number of eggs or in the detection of the presence of the infection^13^.

In addition to the prevalence of *Schistosoma mansoni* infection, possible risk factors for the disease were observed. These factors were divided into social, biological, and behavioral categories, and were collected through the application of a semi-structured questionnaire to each study participant.

### Malacological Survey

Snails were collected between the months of February and July 2019. For the collection, a sketch provided by the Health Department of the municipality was used, identifying the existing water collections in the city. All the collections registered in the sketches were checked.

Obtaining snails in each water collection included the use of a malacological ladle, tweezers, and plastic sieves. A sampling effort was allocated with an average of 10 minutes per collector. The snails collected were packed in properly identified collecting pots and sent to the laboratory, where they were identified according to species and examined for infection by *S. mansoni*. The detection of the infection was performed by the artificial photostimulation technique. Snails that were diagnosed as negative upon the first examination were photostimulated 3 more times, with an interval of one week between each photostimulation. At the end of the fourth photostimulation, the snails were crushed between glass plates to confirm the negative diagnosis for *S. mansoni* infection. In addition, about 10% of the snails in each collection were dissected to confirm the species by analyzing morphological aspects of the reproductive and renal apparatus^14^.

###  Spatial distribution of cases and vectors of *Schistosoma mansoni*

The human cases and the presence of *S. mansoni* vectors were georeferenced by the Global Positioning System (GPS) with a Garmin eTrex 20 device (Garmin Ltd., Schaffhausen, Switzerland). The collection points were entered into the free software Qgis, version 3.18.28 (QGIS Development Team; Open Source Geospatial Foundation Project), where risk maps were built through kernel density estimation. This analysis makes it possible to highlight areas of risk (hotspots) where there is an overlap and agglomeration of cases in a given area^15^, considering a band with a radius of 300 m. The map of the municipality of Lagoa da Canoa was purchased at the website of the Brazilian Institute of Geography and Statistics (IBGE) (https://www.ibge.gov.br/).

### Data Analysis

The data from the questionnaires were tabulated in an Excel spreadsheet, version 2010, and double entry and correction was performed to ensure correct entry of the field data. Parasitic infection was considered the dependent variable and the others were considered as independent variables. The prevalence ratio was estimated directly through Poisson Regression with robust variance adjustment^16^, and the significance level was set at 0.05. The analyses were performed in the Statistical Package for the Social Sciences (SPSS) program, version 26 (IBM Corp., Armonk, NY, USA).

### Ethical considerations

This study was conducted in accordance with the latest version of the principles of the Declaration of Helsinki and was approved by the Ethics Committee for Research involving Human Beings of the Federal University of Alagoas under protocol nº 3827540 (CAEE: 58695716.1.0000.5013).

## RESULTS

### Parasitological Survey

A total of 500 individuals were registered to participate in the study. Of these, the sample studied consisted of 347 individuals from all over the city, 174 (50.1%) individuals from the urban area and 173 (49.9%) from the rural area. Regarding gender, 189 (54.4%) were female and 158 (45.5%) male. Regarding gender and area of residence, the data of this study showed no statistically significant difference with the population data (gender: *x* ^*2*^ 1.789, p = 0.1811; area of residence: *x* ^*2*^ 0.001, p = 0.9779), showing, therefore, that the study sample is representative of the population of the city studied.

Of the 347 individuals participating in the study, 106 (30.5%) were positive for *Schistosoma mansoni* infection. Of the positive individuals, 68.9% (73/106) lived in the urban area, 31.1% (33/106) were in the 39-56 year old age group, 50.9% (54/106) were male, 44.3% (47/106) were married, 66.9% (71/106) had studied until primary school, 97.1% (103/106) had lived in the city for more than 10 years, 57.5% (61/106) were natives of the city, 87.7% (93/106) owned their own house, 84.9% (90/106) earned up to a minimum wage, and 53.8% (77/106) of the participants were farmers.

A significant association was found between the variable of parasitic infection and the variables of areas of residence, profession, fishing in reservoirs or ponds, destination of domestic sewage, number of rooms, and people in the household, ratio room per person, more intense degree contact with water of weirs, dams, and ponds, and contact with water near the home during summer (Table 1, Table 2 and Table 3).

**TABLE 1:** Association of *Schistosoma mansoni* infection with biological and social factors of individuals from the city of Lagoa da Canoa, Alagoas. 2020.

Variables	Frequency (%)	Infection (%)	PR
			(95%CI)
**Area of residence**			
Rural	173 (49.9)	33 (19.1)	1
Urban	174 (50.1)	73 (41.9)	2.25 (1.59-3.20)
Age group (years)			
05 - 22	105 (30.3)	21 (20.0)	1
22 - 39	87 (25.1)	29 (33.3)	1.43 (0.82-2.49)
39 - 56	94 (27.1)	33 (35.1)	1.39 (0.72-2.66)
56 - 73	50 (14.4)	20 (40.0)	1.51 (0.72-3.16)
73 - 90	11 (3.2)	3 (27.3)	0.94 (0.24-3.68)
Gender			
Female	189 (54.5)	52 (27.5)	1
Male	158 (45.5)	54 (34.2)	1.28 (0.94-1.74)
Marital status			
Single	156 (45.0)	36 (23.1)	1
Married	130 (37.5)	47 (36.1)	1.22 (0.74-2.04)
Widower	13 (3.7)	3 (23.1)	0.98 (0.33-2.95)
Divorced	13 (3.7)	4 (30.8)	0.86 (0.34-2.18)
Others	35 (10.1)	16 (45.7)	1.31 (0.78-2.20)
Education			
Elementary school	239 (68.9)	71 (29.7)	1
No formal education	40 (11.5)	12 (30.0)	0.94 (0.60-1.47)
High school or university education	68 (19.6)	21 (30.9)	1.10 (0.72-1.67)
Time living in city			
Up to 1 year	10 (2.9)	2 (20.0)	1
>10 years	337 (97.1)	103 (30.5)	0.99 (0.29-3.37)
Hometown			
No	155 (44.7)	44 (28.4)	1
Yes	192 (55.3)	61 (31.8)	1.19 (0.87-1.63)
Own home			
No	30 (8.6)	12 (40.0)	1
Yes	317 (91.4)	93 (29.3)	1.49 (0.90-2.45)
Monthly family income			
Up to 1 minimum wage	303 (87.3)	90 (29.7)	1
>1 minimum wage	44 (12.7)	15 (34.1)	1.00 (0.61-1.66)
Profession			
Another profession	137 (39.5)	29 (21.2)	1
Farmer	210 (60.5)	77 (36.7)	1.52 (1.03-2.24)

Legend: PR (Prevalence Ratio).

**TABLE 2:** Association of *Schistosoma mansoni* infection with work and leisure activities of individuals from the city of Lagoa da Canoa, Alagoas. 2020.

Variables	Frequency (%)	Infection (%)	PR (95%CI)
**Use weirs, dams, and ponds water for personal hygiene or leisure**			
No	306 (88.2)	89 (29.1)	1
Yes	41 (11.8)	17 (41.5)	1.04 (0.63-1.73)
Use weirs, dams, and ponds water for washing clothes or cars, or bathing animals			
No	233 (67.1)	66 (28.3)	1
Yes	114 (32.9)	40 (35.1)	0.87 (0.57-1.34)
Removing sand of weirs, dams, or ponds			
No	327 (94.2)	103 (31.5)	1
Yes	20 (5.8)	3 (15.0)	0.35 (0.12-1.01)
Working in the field			
No	202 (58.2)	55 (27.2)	1
Yes	145 (41.8)	51 (35.2)	0.89 (0.59-1.36)
Fetching water from weirs, dams, and ponds			
No	276 (79.5)	81 (29.3)	1
Yes	71 (20.5)	25 (35.2)	1.04 (0.67-1.61)
Fishing in weirs, dams, and ponds			
No	321 (92.5)	91 (28.3)	1
Yes	26 (7.5)	15 (57.7)	2.06 (1.34-3.16)
Crossing water of weirs, dams, and ponds			
No	326 (93.9)	99 (30.4)	1
Yes	21 (6.1)	7 (33.3)	0.93 (0.50-1.70)
Degree contact with water of weirs, dams, and ponds			
No contact	144 (41.5)	34 (23.6)	1
More Intense	102 (29.4)	39 (38.2)	1.87 (1.04-3.38)
Less Intense	101 (29.1)	33 (32.7)	1.38 (0.79-2.41)

Legend: PR (Prevalence Ratio).

**TABLE 3:** Association of *Schistosoma mansoni* infection with factors related to home and peridomicile in individuals from the city of Lagoa da Canoa, Alagoas. 2020.

Variables	Frequency (%)	Infection (%)	PR (95%CI)
**Water supply**			
Public	245 (70.6)	75 (30.6)	1
Water well or cacimba-type well	102 (29.4)	31 (30.4)	0.92 (0.63-1.33)
Drinking water treatment			
No	169 (48.7)	56 (33.1)	1
Yes	178 (51.3)	50 (28.1)	0.98 (0.71-1.35)
Destination of domestic sewage			
Open air sewage	145 (41.8)	32 (22.1)	1
Public sewage system or latrine	202 (58.2)	74 (36.6)	1.76 (1.22-2.55)
Destination of garbage			
Dumped in the surrounding or burned	45 (13.0)	8 (17.8)	1
Municipal public collection	302 (87.0)	98 (32.4)	1.79 (0.89-3.31)
Sanitary installation at home			
No	11 (3.2)	4 (36.4)	1
Yes	336 (96.8)	102 (30.3)	0.90 (0.35-2.32)
Flooring in households			
Masonry flooring	337 (97.1)	102 (30.3)	1
Wood	10 (2.9)	4 (40.0)	0.99 (0.39-2.52)
Number of people in the household			
Up to 5 people	290 (83.6)	86 (29.6)	1
>5 people	57 (16.4)	20 (35.1)	2.03 (1.22-3.37)
Number of rooms in the household			
>5 rooms	156 (45.0)	38 (24.5)	1
Up to 5 rooms	191 (55.0)	68 (35.6)	1.89 (1.2-2.79)
Ratio room per person			
<1 room/person	59 (17.0)	17 (28.8)	1
>1 room/person	288 (83.0)	89 (30.9)	1.81 (1.10-2.97)
Paved street			
No	192 (55.3)	56 (29.2)	1
Yes	155 (44.7)	50 (32.2)	1.12 (0.82-1.54)
Accumulation of water at home in the summer			
No	304 (87.6)	89 (29.3)	1
Yes	43 (12.4)	17 (39.5)	1.07 (0.59-1.94)
Accumulation of water at home in the winter			
No	270 (77.8)	78 (28.9)	1
Yes	77 (22.2)	28 (36.3)	1.04 (0.63-1.72)
Contact with water in the peridomicile during summer			
No	286 (82.4)	81 (28.3)	1
Yes	61 (17.6)	25 (40.9)	1.61 (1.06-2.44)
Contact with water in the peridomicile during winter			
No	196 (56.5)	57 (29.1)	1
Yes	151 (43.5)	49 (32.4)	1.08 (0.73-1.58)

Legend: PR (Prevalence Ratio).

The intensity of the infection, measured as the number of eggs per gram of feces, had the highest proportion (90.6% [96/106]), in the class of mild intensity, followed by the moderate class (8.5% [9/106 individuals]), and only 0.9% (1/106) of individuals in this study had heavy infection.

### Malacological Survey

From February to June 2019, 792 snails were collected in the city, 600 of which were captured alive and 192 were just shells. Most of the snails, 86.5% (685/792), were collected in the rural area of the municipality, while 13.5% (107/792) were collected in the urban area. Regarding the species of snails collected, 84.1% (666/792) of the snails were *Biomphalaria glabrata* and 15.9% (126/792) snails were *Biomphalaria straminea*. In one locality of the urban area, the two species of snails were found in the same water body. As for the infection of these snails, neither species was found to be infected with *Schistosoma mansoni*.

###  Spatial distribution of human cases and vectors of *Schistosoma mansoni*

**FIGURE 1:**
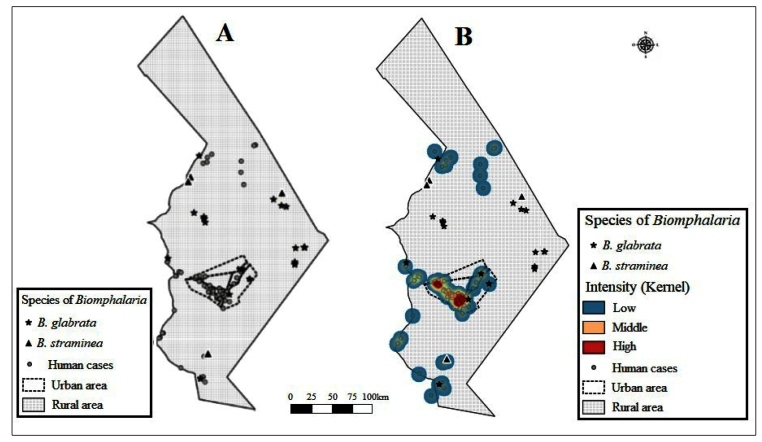
Distribution of human cases and vectors of *Schistosoma mansoni* in the city of Lagoa da Canoa, Alagoas, Brazil. **Legend:** *B. glabrata* (*Biomphalaria glabrata*) and *B. straminea* (*Biomphalaria straminea*).

Figure 1A shows a random spatial distribution of human cases of schistosomiasis and snail vectors of *Schistosoma mansoni*, with the animals predominantly present in the rural area of the city and the human cases concentrated in the urban area. In addition, Figure 1B shows, by means of kernel density estimation, a cluster of cases (hotspots) at two points in the urban area of the city. This figure also shows that in the rural area there was no formation of hotspots for human cases of schistosomiasis in the city of Lagoa da Canoa and that only a single collection point, for the species *B. glabrata*, was close to the human case cluster of the disease.

## DISCUSSION

In Alagoas, studies have shown that schistosomiasis persists as a rural endemic in areas of low prevalence^17^ ^,^ ^18^, which differs from the data found in this study. On the other hand, a high prevalence has already been found in urban areas of two high prevalence cities in the state^19^, but the authors of the study did not perform the investigation, at the time, in the rural area of the municipality. A possible explanation for the high proportion of urban cases diagnosed in this study is the work logistics of the PCE technicians associated with the municipality’s main agricultural activity, cultivation of tobacco^10^.

In the municipality, the PCE campaigns are concentrated in localities of the rural area (personal communication of the technicians of the municipality program, February 2019), where the parasitized individuals are treated, which reduces the positivity rate in these locations in the following campaign. In addition, most of the participants in this study, men and women, work in the cultivation of tobacco in the rural area for most of the year. Thus, the low activity of the PCE campaigns in the urban area may have contributed to the high positivity rate of human cases in this area because these residents are submitted daily to contact with waters possibly containing vector snails of *Schistosoma mansoni*; in this study, it was identified that these snails are predominantly present in the rural area of the city.

The occurrence of two of the three vector species of epidemiological importance in the city reinforces the magnitude of the disease for the municipality. The two species were found both in the urban and rural areas and although these animals were not diagnosed positive for infection by *Schistosoma mansoni*, human infection data point to an epidemiological scenario distinct from the classical scenario, where vectors are found positive for the elimination of cercariae, thus characterizing areas of risk for transmission^20^ ^,^ ^21^. In another study^22^, a focus of urban transmission was identified in Pernambuco, characterized by the presence of *B. straminea* eliminating cercariae in urban areas. In the same study, these authors classified most breeding sites as potential foci, as *Schistosoma mansoni* DNA was found in the tissues of the snails collected at these sites. Another study^23^, also in Pernambuco, showed that of the 64 verified breeding sites of *B. straminea*, 4 (6.25%) of them had snails releasing cercariae when exposed to artificial light, while the infection of 54 (84.4%) could only be determined by using a molecular biology technique. Other studies have also shown that the method of artificial photostimulation of snails is not able to detect infection in these animals^24^ and that molecular techniques are more sensitive in detecting infection of snails by *S. mansoni* ^21^ ^,^ ^25^ ^-^ ^27^.

Given the scenario described here, with studies showing that the method for the diagnosis of infection of *S. mansoni* vectors - artificial photostimulation, which is recommended by the Ministry of Health - is not able to detect infection of snails, it may be worthwhile to consider a reformulation of the guidelines for classification of areas at risk for the transmission of schistosomiasis, because criteria based solely on the elimination of cercariae by snails can exclude from the priority area locations with potential for the transmission of parasitosis.

This study also showed that some classical risk factors for the transmission of *S. mansoni* were not implicated in the occurrence of human cases in the city studied. Using weir water for personal hygiene or leisure, washing clothes or cars, or bathing animals, removing sand, and crossing these waters were some of the factors that were not significant in the association with infection by the parasite. On the other hand, it was seen that for the city studied, living in the urban area, being a farmer and having frequent, close contact with water presented a higher prevalence and were significantly associated with infection by *Schistosoma mansoni*. 

However, it is worth noting that the data obtained with the application of the questionnaires may present some biases that prevent a more accurate analysis from being performed. Memory bias is a limiting factor for epidemiological studies based on the application of data collection instruments, such as questionnaires^28^, which may apply to the present study, and is therefore considered a limiting factor for this study.

On the other hand, the data of the present study allowed some hypotheses to be raised: 1) other localities with characteristics similar to the study area of this study may also present schistosomiasis mansoni concentrated in the urban area of the municipality, and this requires special attention from local health teams; 2) the rate of natural infection of the snails collected in the city of Lagoa da Canoa, Alagoas, diagnosed by a molecular method, would show a higher risk scenario than that presented in this study; and 3) the methodology of diagnosis of human cases used in the Schistosomiasis Control Program does not show the real epidemiological situation of a municipality, which is considered to be of low endemicity.

Here, the reading of two slides was used as the method of analysis for each sample of fecal material collected, which were 3 samples on alternate days. In addition, two analysts read each of the samples, which may have contributed to the increased sensitivity of the method used. Other studies have demonstrated this relationship between the number of slides read and the positivity rate of human cases of schistosomiasis^5^. Thus, these data further reinforce the need for a diagnostic measure that can contribute and increase the efficiency of local actions of schistosomiasis control programs, since the guidelines of the Ministry of Health recommend the collection of a single sample of fecal material, followed by the reading of 2 slides in the areas endemic for the disease^29^.

Other diagnostic methods, such as the search for circulating antigen, detected through urine processing of the possibly infected individual are already being tested in areas of low prevalence for schistosomiasis, but the existence of dubious chromatographic bands regarding the positivity or negativity of the sample for worm antigens means that this method is not recommended, neither for surveys nor for the routine diagnosis of PCE activities^30^ ^-^ ^32^. In this sense, optimizing an existing and in-use method, such as Kato-Katz, is a reasonable measure in the fight against *S. mansoni* infection and this work shows that the way the diagnosis is being made in areas considered to have low prevalence is not adequate to estimate the real magnitude of the disease in these localities.

Therefore, this study presented a new epidemiological scenario for an area endemic for *Schistosomiasis mansoni* infection in the state of Alagoas, comprising the following elements: 1) a high proportion of positive individuals in the urban area of the municipality studied, evidenced by the formation of hotspots in this area; 2) negative results for the pathogen in snails collected in the city; 3) the almost complete absence of breeding sites near the clusters of human cases, evidenced by the greater number of animals collected in the rural area of the municipality; and 4) the non-association between some risk factors, considered classic in the triad of transmission of *Schistosoma mansoni*, and the infected individuals diagnosed in the study.
